# Accurate Reaching after Active But Not Passive Movements of the Hand: Evidence for Forward Modeling

**DOI:** 10.1155/2008/972542

**Published:** 2008-07-15

**Authors:** H. Branch Coslett, Laurel J. Buxbaum, John Schwoebel

**Affiliations:** ^1^University of PennsylvaniaPhiladelphiaPAUSA; ^2^Moss Rehabilitation Research InstitutePhiladelphiaPAUSA; ^3^Thomas Jefferson UniversityPhiladelphiaPAUSA; ^4^Cazenovia CollegeCazenoviaNYUSA

**Keywords:** Forward model, parietal, proprioception, pointing, efference copy, sensory loss

## Abstract

Converging behavioral findings support recent models of motor control suggesting that estimates of the future positions of a limb as well as the expected sensory consequences of a planned movement may be derived, in part, from efference copies of motor commands. These estimates are referred to as forward models. However, relatively little behavioral evidence has been obtained for proposed forward models that provide on-line estimates of current position. We report data from a patient (JD) who reached accurately to visualized targets with and without vision of her hand despite substantial proprioceptive loss. Additionally, we administered a double-start reaching test to examine the possibility that efference copy information could be used to estimate current limb position. JD reached accurately, without vision, to a final target after actively reaching to a landmark, but exhibited severely impaired reaching after passive movements to the landmark. This finding suggests that forward modeling of efference copy signals may provide relatively accurate estimates of current limb position for the purpose of motor planning. The possibility that such estimates may also contribute to the awareness of body position and to self-recognition is discussed.

## 1. Introduction

"You are here". ↗

This information on trail-head maps as well as mall directories provides people with critical data when trying to decide how far and in what direction they need to move in order to reach their destination. As suggested by recent models of motor control, similar information is also required in order to accurately reach toward an object (see [7,37]). That is, it is essential to have accurate knowledge of the current position of the hand/limb in order to accurately plan and/or modify movements toward a target. While estimates of limb position may be derived from visual and proprioceptive feedback, several lines of evidence suggest that copies of motor commands (i.e., efference copies) may be used to estimate the current and future positions of a limb as well as the sensory consequences of planned movements (e.g. [1,6,8,9,16,25]). Such estimates are referred to as forward models since they are thought to be internal models of the consequences of motor commands. Two of the proposed functions of forward modeling are: (1) to increase the accuracy of estimates of the current position of a limb beyond that possible based on sensory information alone [40] and (2) to provide corrective feedback regarding the accuracy of the predicted outcome of a motor command without the delays (estimated to range from 100–250 ms; e.g., see [17]) that occur with sensory feedback [11].

ISSN 0953-4180/08/$17.00 © 2008 – IOS Press and the authors. All rights reserved

Behavioral evidence consistent with the latter proposed function of forward modeling has primarily been derived from experiments involving a double-step paradigm. In this paradigm, participants are typically asked to look at and point to a peripheral visual target without being able to view their moving limb. On a subset of trials, the target changes location during the saccade toward the target and thus requires participants to alter the trajectory of their hand in order to accurately reach toward the new target location. Critically, changes in the trajectory of the hand on these double-step trials have been observed to occur with latencies as short as 30 ms; i.e., a latency too short to be derived from sensory feedback [35]. Further support for this proposed function of forward modeling has also been obtained in the study of a deafferented patient (GL) using a similar paradigm [1]. Taken together, the ability to correct movement trajectories prior to (or absent of) the availability of visual and proprioceptive feedback provides strong support for the role of forward modeling in predicting the outcome of motor commands and providing on-line corrective feedback concerning the discrepancy between this outcome and the target location.

Several lines of evidence are consistent with the possibility that forward modeling is involved in predicting the endpoint of a reaching movement, however there is relatively little behavioral evidence concerning the possibility that forward modeling is involved in estimating the current position of a limb, which would provide essential information for motor planning. One observation consistent with this proposed function was reported by Wolpert et al. [40], who required participants to estimate the location of their unseen hand after moving a manipulator that could apply both resistive and assistive forces to the hand. The observed errors in these estimates were better predicted by an engineering model (i.e., Kalman filter) that integrates both efference copy and sensory feedback information than by models utilizing only one of these sources of information. Consistent with this finding, Ferrer et al., (2003) reported that a deafferented patient (GL) was able to detect angular disparities between the direction of her own unseen hand movements and those of a computer generated virtual hand which she viewed in a mirror positioned directly above her own hand (i.e., so that it appeared as if she was viewing her own hand). While these investigations suggest that forward modeling is involved in estimating the current position and movement of a limb, they do not directly address the possibility that forward modeling of efference copy signals may help to inform the generation of a subsequent motor plan.

The goal of the present investigation is to further explore the possibility that forward modeling based on efference copy information may play a role in estimating current hand position and informing new motor plans. We reasoned that evidence of accurate reaching in the context of degraded or absent proprioceptive or visual information concerning hand position would suggest that an estimate of initial hand position could be maintained based on the efference copy information concerning the prior hand movement. We examined this possibility in a patient (JD) with profound sensory loss after a stroke. First, we assessed whether JD could plan and execute accurate reaches to visual targets (Study 1) and reach accurately based on non-visual feedback (Study 2). Study 3 was designed to replicate previous findings suggesting impaired proprioception in JD and to further explore the possibility that JD is able to use forward modeling of efference copy information in order to estimate initial hand position and plan reaching movements. JD was asked to perform pointing movements, without vision, in a double-start paradigm. On each trial, JD's hand was either actively or passively moved to a landmark before she pointed to a final target. We predicted that if JD was able to estimate the location of her hand at the landmark location, based on forward modeling of efference copy information, then her reach to the final target would be more accurate after the active as compared to the passive movements to the landmark, since efference copy information would not be likely during passive movements.

## 2. Participant & background testing

JD, a college educated 77-year-old right handed female, suffered a stroke approximately 3 years prior to testing. Neurologic examination revealed a right hemiplegia; she was able to elevate her right shoulder and weakly flex and extend her leg at the hip but there was no voluntary movement of the distal arm or leg. She was confined to a wheelchair. Sensory examination revealed a substantial elevation in sensory threshold to pin, light touch and temperature on the right and a mildly elevated sensory threshold on the left side of her body. She exhibited a substantial impairment in localizing tactile stimuli that she acknowledged feeling; she often erred, for example, in reporting whether she had been touched on the arm or the leg. Proprioception was assessed by asking JD to determine if a body part was moved by the examiner and, if so, to indicate the direction of the movement; testing was performed with her eyes closed and extremities relaxed. JD was able to detect large amplitude movements (e.g., $>30^\circ$) of the right wrist, elbow and shoulder but was at chance in determining the direction of the movement. Performance was somewhat better on the left; she was able to determine if the fingers of her left hand were passively moved but was unable to determine the direction (up or down) of the movement; a similar pattern of impairment was observed at the left wrist. She could discriminate the direction of passive movements of approximately 15° involving her left shoulder but failed to identify the direction of less substantial movements. Despite her striking sensory deficits, JD reached to touch visualized targets accurately with her left hand. There was no neglect or optic ataxia. There was no evidence of peripheral neuropathy; reflexes were present throughout and pathologically brisk on the right. There was no distal to proximal gradient of her sensory loss. Studies for unusual causes of a sensory neuronopathy (e.g., diabetes, syphilis, metabolic impairments, paraneoplastic syndrome) were negative.

CT scans indicated infarction of the left posterior temporal, lateral occipital, and inferior parietal lobe; the lesion extended into the white matter of the left hemisphere and appeared to undercut the left superior parietal lobule and, perhaps, disrupted descending motor fibers. White matter lucencies were noted in both hemispheres and there was generalized atrophy. A lateral view of the left hemisphere on which the extent of the cortical lesion is shown in Fig. 1. The investigations were approved by the Temple University Institutional Review Board and were conducted in accordance with the Declaration of Helsinki after informed written consent was given.

Language was assessed with the Boston Diagnostic Aphasia Examination [13]. She exhibited a fluent aphasia with frequent paraphasic errors; comprehension was moderately impaired and repetition was relatively spared. Performance was most consistent with a diagnosis of transcortical sensory aphasia. As described in Buxbaum et al. [5], JD exhibited a severe ideomotor apraxia with gross spatio-temporal errors; scores ranged between 7–30% correct on the Florida Apraxia Screening Test [27]. There was no evidence of neglect on the star cancellation or figure copying tasks from the Behavioral Inattention Test [36] and she performed normally on a modified version of the "fluff test" (Jehkonen, unpublished) designed to assess body neglect.

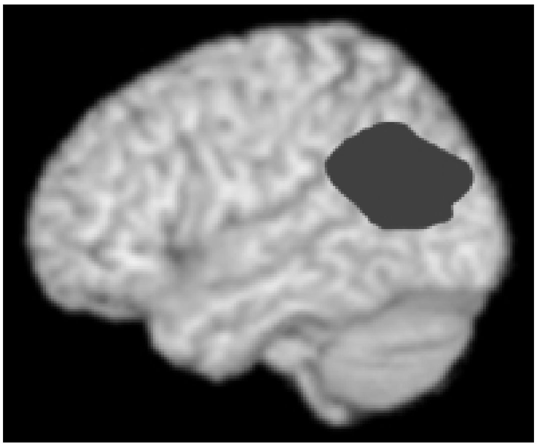

Fig. 1. A lateral view of the left hemisphere depicting the extent of JD's lesion.

JD also exhibited autotopagnosia [24]. She was unable to point to named body parts on either her body (4/20 correct) or the body of the examiner (2/20 correct). She was also impaired on non-verbal tasks assessing her body knowledge; she was unable to point to the same parts on herself that the examiner pointed to on himself (6/20 correct). Following Sirigu et al. [32], we (e.g., Schwoebel and Coslett, 2005) have distinguished between three putative types of body representations: the "body image", or conceptual knowledge about the body including knowledge of the function of body parts; the "body structural description", a representation of the shape and location of body parts on oneself and others; and the "body schema", a dynamic, on-line, real-time representation of the relative positions of body parts. As noted in Schwoebel et al. [29], JD performed poorly on tasks assessing all three types of body representations.

JD's ability to discriminate the location of tactile stimuli was assessed in a series of tasks in which a suprathreshold tactile stimulus was delivered to a number of different locations on the right or left side of her body (see [29] for details). In one task, she was asked to determine if two tactile stimuli were delivered to the same or different location; she performed at chance in determining whether successive tactile stimuli were presented in the same location or a location 4 cms. away. In a second series of experiments, JD was asked to touch a location on her body surface that had been touched by the examiner. She made frequent errors, particularly for stimuli on the right side of her body.

Finally, JD performed well on a number of challenging "executive function" tasks indicating that her ability to solve problems and follow complex instructions was at least relatively preserved. For example, she performed normally on the five-move problems from the Tower of London test (M. Schwartz, unpublished data).

## 3. Study 1: Reaching with vision

JD exhibits striking deficits in multiple sensory modalities and performs poorly on a variety of tasks assessing different types of body representations. Despite these deficits, clinical assessment suggested that she was able to reach accurately to visualized targets. The first study was performed to assess her reaching behavior more formally. To this end, she was asked to make precise movements that required not only transport of the hand to a target but also rotation of the arm at the wrist, elbow and shoulder.

### *3.1. Design & procedure*

JD was asked to perform a version of the card-posting task designed by Goodale et al. [12]. JD was instructed to insert her hand into a 5 cm. by 15 cm. slot that was cut into the center of a 2 cm. thick circular board that was 60 cm. in diameter. The board was held upright, so that the slot was facing JD and located 30 cm. in front of her chest in her midline. The orientation of the slot was either vertical, 45° to the left, 45° to the right, or horizontal. Each of these orientations was presented nine times resulting in a total of 36 randomly ordered trials. On each trial, JD was asked to move her left hand from its starting position resting on the table in front of her until her fingers were inserted into the slot. Reaches were recorded as accurate if JD inserted her fingers into the slot without contacting the edges of the slot or the surrounding board.

### *3.2. Results & discussion*

JD performed perfectly on this task. Further, she appeared to reach toward the slot in a smooth motion without making any awkward adjustments to the trajectory or the orientation of her hand. JD's performance on this task has important implications for the interpretation of the main findings reported in this paper. Specifically, her lesion does not appear to have disrupted her ability to process visual information concerning target location and orientation or her ability to generate and execute accurate motor plans.

## 4. Study 2: Reaching without vision

JD's good performance in Study 1 suggests that she is able to generate and execute a precise motor plan despite her lack of proprioception. As she was tested with her eyes open, however, it is possible that she achieved good results by employing a visually based compensatory strategy. More specifically, JD may have relied on visual feedback to determine the discrepancy between her arm and the target and employed this information in an iterative fashion to correct her movement trajectory and arm rotation. The fact that she appeared to perform the task normally with respect to the timing of movement initiation and execution argues against but does not eliminate this possibility. The following experiment was performed to assess her ability to move without being able to view her moving limb.

### *4.1. Design & procedure*

JD was asked to reach toward and touch a nine watt light bulb target in two conditions that either allowed vision of her limb throughout each trial (i.e., visual trials) or obscured vision of her limb, but not the target, throughout each trial (i.e., non-visual trials). For the non-visual condition, JD wore goggles that filtered all frequencies except near infrared light; while wearing these goggles she could not see her hand but could see the filament of the bulb that served as the target. The goggles were removed for the visual trials. On each trial, the visual target was positioned at one of six possible locations on the table in front of JD. Targets were approximately 46 cm. in front of JD and were located 15, 30, or 45 cm. to the left of her midline or 15, 30, or 45 cm. to the right of her midline. On each trial, JD was instructed to move her left hand from its starting position on the table in front of her and to touch the target with her index finger. Pointing accuracy was measured as the distance from the point on the table surface at which JD made initial contact with her index finger and the target position.

There were two blocks of 30 trials for both the visual and non-visual trials; one block of each type of trial was administered in each of two sessions using an ABBA design. Trials were separated by approximately five seconds.

### *4.2. Results & discussion*

JD performed perfectly except for the second (3 cm. error to left of target) and third (2 cm. error to left of target) trials of the first block of non-visual trials. That is, she executed smooth reaches toward the target and her index finger initially contacted the target on all but two trials. Further, on the non-visual trials, she was not observed making any awkward adjustments in her reaches that would have been consistent with a strategy of occluding the visual stimulus with her hand in order to improve pointing accuracy. There was no significant difference between pointing errors in the visual (0 cm. mean error) and the non-visual (0.17 cm. mean error) conditions, $t(29) = 1.41,\ p = 0.17$.

Consistent with the findings of Study 1, these findings suggest that JD is able to process visual information concerning target location and generate and execute accurate motor plans. Furthermore, these findings demonstrate that JD is able to use proprioceptive and/or forward model information to guide her reaches in the absence of visual feedback. Importantly, even if no online adjustments to the motor plan were required during her reaching movements, information concerning the starting position of her left hand would be essential to the formation of an efficient motor plan. Since visual information concerning initial hand position was eliminated in the non-visual trials of Study 2, we argue that JD's accurate reaching movements on these trials were based on non-visual feedback that included information concerning the initial position of her limb.

## 5. Study 3: Reaching in a double-start task

Although she exhibited significant proprioceptive deficits involving her left hand, JD was able to reach to targets accurately without visual feedback. One possible explanation for her accurate reaching despite impoverished sensory feedback regarding hand position is that JD utilized efference copy to generate a forward model that provided information about the current state of the limb. To test this hypothesis, JD was asked to reach to a target with her eyes closed in two different conditions. In one condition, she moved her hand to touch a landmark before reaching from the landmark to touch the target. In the second condition, her hand was passively moved from its resting position to the same landmark, from which she initiated a reach to the target. We reasoned that if efference copy generates a forward model that provides information about the anticipated motor consequences of motor plans, she should reach accurately after an active movement of her hand to the landmark. In contrast, we predicted that she would reach inaccurately after her hand had been passively moved to the landmark because she would have no efference copy to inform her motor system of the new location of her hand and her proprioceptive deficit would preclude accurate updating of the position of her hand during and after the passive movement.

### *5.1. Design & procedure*

JD was asked to point to the nine watt light bulb target in a double-start paradigm. To prevent visual feedback, after foveating and verbally indicating her perception of the target, JD was asked to close her eyes and to keep her eyes closed throughout each trial; her eyes were closed prior to and during the movement (active or passive) of her hand to the landmark and from the landmark to the target. In the active movement condition, JD was instructed to move her hand from its resting position on her left thigh to one of two possible landmarks before pointing to the final target (i.e., the lightbulb). That is, she was asked to move so that her index finger contacted either her nose or a round disk 2 cm. in diameter that was located on the table 10 cm. in front of her in the midline. In the passive movement condition, JD's hand was passively moved by the experimenter to one of the landmarks, from which she reached to the final target. We selected the tactile (nose) and external (disk) as landmarks in order to examine the possibility suggested by previous findings that tactile information may help to improve position estimates during passive but not active movements [23]. We expected better pointing accuracy after passive movements to the nose than to the disk.

There were three target locations. A central target location was 51 cm. in front of JD in her midline and two additional locations were 15 cm. to the left and right of the central target. Testing occurred in a blocked ABBA design (Active Movement to Nose, Passive Movement to Nose, Passive Movement to Disk, Active Movement to Disk) with 21 trials in each block (seven trials per target location) resulting in a total of 84 randomly ordered trials. Pointing accuracy was measured as the distance between the point on the table surface at which JD made initial contact with her index finger and the final target position. Four age and education matched controls were also tested.

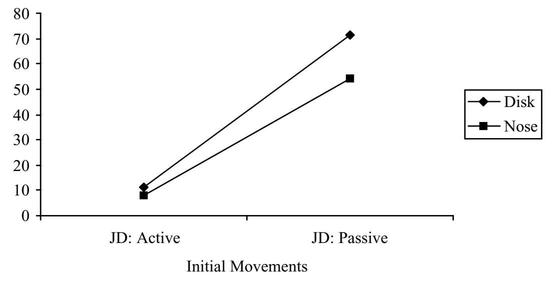

Fig. 2. Mean pointing errors in the double-start task in Study 3.

### 5.2. Results

Mean errors for the active and passive conditions for both the nose and disk landmarks are presented in Fig. 2. As there was no difference in accuracy between the three target locations, data were collapsed across this variable in all subsequent analyses. A 2 (Initial Movements: Active vs. Passive) X 2 (Landmarks: Disk vs. Nose) repeated measures ANOVA revealed a significant main effect of initial movement, $F(1,20) = 784.61,\ p < 0.001$, indicating greater pointing accuracy following active (Mean error 9.60 cm.) as compared to passive (Mean error 62.83 cm.) initial movements. There was also a significant main effect of landmark, $F(1,20) = 25.09,\ p < 0.001$, indicating JD's greater pointing accuracy following initial movements to the nose (Mean error 31.00 cm.) as compared to the disk (Mean error 41.43 cm.). However, interpretation of these results was modified by the finding of a significant interaction between initial movement and landmark, $F(1,20) = 12.71,\ p < 0.002$.

Planned comparisons revealed that the advantage for initial movements to the nose as compared to the disk approached significance when JD was making active movements, $t(20) = 2.02,\ p < 0.06$, and was significant during the passive movement condition, $t(20) = 4.79,\ p < 0.001$. However, consistent with the significant interaction between initial movement and landmark reported above, the advantage was significantly greater during the passive than the active movement condition, $t(20) = 3.57,\ p < 0.002$.

The same repeated measures analysis of the mean pointing accuracy data for controls indicated no significant main effects or interactions (Fs $<$ 1). Mean pointing errors were 2.48 cm. and 2.40 cm. after active movements to the nose and disk respectively and 2.62 cm. and 2.55 cm. after passive movements to the nose and disk respectively. Although JD was significantly more accurate in the active as compared to passive movement conditions, she was significantly less accurate in the active movement conditions than control participants ($t(40) = 5.45,\ p < 0.001$ and $t(40) = 6.07,\ p < 0.001$ for movements to the nose and disk respectively).

Visual inspection of her reaching behavior revealed striking differences between the active and passive conditions. In the former condition, her movements appeared to be quick, confident and at least relatively precise. In contrast, in the passive movement conditions, she exhibited random, groping movements; indeed, she often stated that she had lost track of the location of her hand. Consistent with this claim, when reaching from the disk to the target, a distance of approximately 41 cm, her mean error was 71.5 cm, reflecting the fact that she made large amplitude, sweeping movements of arm in search of the target.

### 5.3. Discussion

The main finding of the present investigation is that despite impoverished proprioception, JD was able to point at least relatively accurately, without vision, after actively pointing to a landmark. Consistent with recent models of motor control (e.g. [7,38]), these data strongly suggest that estimates of current hand location may be derived from efference copy signals and that this estimate may, in turn, play a role in the generation of accurate motor plans for reaching. Furthermore, JD's catastrophic failure on the passive movement trials but relatively accurate reaching on active trials suggests that the information regarding the current position of the hand provided by the forward model is at least relatively precise. The fact that JD is less accurate than controls even in the active movement condition is consistent both with classic two-stage models and more recent accounts of motor control. Two-stage models of reaching postulate that sensory feedback, which is impoverished for JD, is important in minimizing error in the later stages of movement [17,41]. More recent models of motor control (e.g. [40]) assume that optimal performance is achieved by integrating sensory and forward model estimates of limb position.

A second significant effect is that JD's reaching accuracy improved following both active and passive movements to her nose as compared to the disk. This finding is consistent with data from a previous investigation with normal participants [23]. We suggest that the additional tactile information provided when JD's index finger contacted her nose may have been integrated with efference copy information in the active movement condition in order to allow for a more precise estimate of hand location and may have partly compensated for her impaired proprioception in the passive movement condition. While this account requires further investigation, it is interesting to note that it is at least consistent with dramatic illusions suggesting interactions between tactile, proprioceptive, and visual information (e.g. [3,20,33]).

An alternative but not mutually exclusive possibility is that her nose serves as a spatial anchor. Although JD exhibited a substantial impairment in the ability to localize stimuli delivered to her body surface, even a coarse coding of the position of her body in space may have provided more precise spatial information regarding her starting point than the disk located in extracorporeal space.

Finally, we note that there is one alternative interpretation of the data that should be addressed. In the passive movement condition JD may have deferred programming the movement from the intermediate location (disc or nose) until she arrived at that location whereas in the active condition she may have programmed a single movement that incorporated two segments (to the intermediate location and from there to the target). In the former condition she would have been required to retain an accurate representation of target position for a longer duration before programming the movement; on this argument, her deficit might be attributed to a failure to maintain a spatial representation of target location. The possible effect of memory decay on JD's performance was addressed in experiments (Studies 5 and 6) described in Schwoebel et al. [29]. In those studies, JD was asked to point to remembered target positions after a delay of 3 seconds and, in another condition, after her arm had been passively moved and returned to its original location. In both conditions, her performance did not differ from the immediate reach condition, suggesting that neither impaired memory for target location nor passive movement of her limb can account for her dramatic deficit on this task.

## 6. General discussion

Data from JD suggest that a forward model may play an important role in providing an updated, real-time representation of the position of the body in space; this representation has been termed the "body schema" in the neurologic literature and the "updated state estimate" in one recent model of motor planning and control [37]. Evidence for the normal maintenance of internal estimates of limb position has been provided by a demonstration of its absence in a patient (PJ) with a left parietal lobe lesion [39]. When asked to indicate, without vision, the perceived position of her right immobile hand by pointing at it with her left hand, PJ was accurate until about 18 seconds had elapsed, at which point her estimates started to become increasingly inaccurate. While the present finding does not address JD's ability to maintain position estimates over time, it does suggest that she may be able to at least briefly maintain an estimate derived from efference copy (and perhaps tactile) information in order to plan the final pointing movement in the double-start pointing paradigm.

It is difficult to precisely identify the anatomical areas associated with JD's pattern of performance in the double-start task due to the extent of the lesion and the presence of bilateral white matter lucencies. However, a growing body of neurophysiological and neuroimaging evidence suggests that the parietal cortex consists of several functional subdivisions that include areas involved in the processing of proprioceptive and efference copy information [18,21,22,30]. Furthermore, it has been suggested that there may be some functional differentiation between the processing of sensory and efferent signals such that anterior regions appear to play a greater role in the processing of sensory/proprioceptive signals while posterior parietal cortex plays a greater role in the processing of efference copy signals and in the planning of movements [4,14]. Thus, it may be that JD's parietal lesion may have disrupted her ability to process proprioceptive information while sparing regions involved in receiving efference copy information and generating forward models based on this information. Converging evidence from TMS, neuropsychological, and neuroimaging investigations suggests that the posterior parietal cortex may play a role in generating forward models for the purpose of predicting the end-point of an ongoing movement that allows for on-line corrective feedback during reaching [6,8,16,25]. Furthermore, as noted in the introduction, behavioral evidence and recent models of motor control suggest that, in addition to a forward model that predicts the future position of a limb, there may be forward models that predict the sensory consequences of movements and, as suggested by the current findings, forward models involved in estimating current position [2,19,37,38]. Further research will be necessary in order to identify the precise anatomical substrates of these additional putative forward models. However there is compelling behavioral and neuropsychological evidence suggesting that estimates of current position and movement may also depend upon the processing of efference copy information in the parietal cortex and that, in addition to playing a role in motor planning, these estimates may also contribute to the awareness of body position and movement and to a sense of agency or self-recognition [10,31,34]. Indeed, investigations of patients with amputated limbs suggest that such an estimate may even produce a "You are here" signal regarding the position and movement of a limb that no longer exists [11,26].

A final point that warrants mention is the fact that JD exhibited substantial proprioceptive loss on the left side after a left hemisphere lesion. There are several possibilities for this. CT scan revealed some degree of deep white matter abnormalities in both hemispheres that may have contributed to this finding. Alternatively, the scan could have missed a small brainstem infarct that disrupted proprioceptive input to the right hemisphere. A third possibility is that for JD the high level representation of body in space, the body schema, was lateralized to the left hemisphere. In a large study of body representations in subjects with unilateral lesions, we [28] observed body schema deficits that involved bilateral arms and legs even in some subjects with good quality imaging. We suggest that the element of the body schema of which subjects can be aware and supports explicit verbal responses may be unilaterally represented in some subjects. This conjecture will require further investigation.

## Acknowledgments

This research was supported by RO1 NS048130 and RO1 NS037920 to HBC and RO1 NS036387 to LB. We would like to thank Dr. Steven Jax for his helpful comments.
